# Thermodynamic Simulation of Carbonate Cements-Water-Carbon Dioxide Equilibrium in Sandstone for Prediction of Precipitation/Dissolution of Carbonate Cements

**DOI:** 10.1371/journal.pone.0167035

**Published:** 2016-12-01

**Authors:** Yiping Duan, Mingshi Feng, Xinyan Zhong, Ruishu Shang, Lihong Huang

**Affiliations:** 1 Department of Chemical and Pharmaceutical Engineering, Chengdu University of Technology, Chengdu, China; 2 State Key Laboratory of Oil and Gas Reservoir Geology and Exploitation, Chengdu University of Technology, Chengdu, China; 3 Richard G. Lugar Center for Renewable Energy, Indiana University-Purdue University, Indianapolis, IN, United States; East China Normal University, CHINA

## Abstract

Carbonate cements, such as calcite, dolomite, ferrocalcite and ankerite, play important roles in the formation of pores in sandstones: precipitation of carbonate cements modifies pores and inhibits compaction, while dissolution creates secondary pores. This work proposed a precipitation-dissolution model for carbonate cements-CO_2_-H_2_O system by means of ion equilibrium concentration ([M^2+^], M = Ca, Mg, Fe or Mn) with different factors, such as temperature, depth, pH, $P_{{\text{CO}}_{2}}$, variable rock composition and overpressure. Precipitation-dissolution reaction routes were also analyzed by minimization of the total Gibbs free energy (Δ*G*). Δ[M^2+^], the variation of [Ca^2+^], [Fe^2+^], [Mg^2+^] or [Mn^2+^] for every 100 m of burial depths, is used to predict precipitation or dissolution. The calculation results indicate that the increasing temperature results in decrease of equilibrium constant of reactions, while the increasing pressure results in a relatively smaller increase of equilibrium constant; As a result, with increasing burial depth, which brings about increase of both temperature and pressure, carbonate cements dissolve firstly and produces the maximal dissolved amounts, and then precipitation happens with further increasing depth; For example, calcite is dissolving from 0.0 km to 3.0 km with a maximal value of [Ca^2+^] at depth of 0.8 km, and then precipitates with depth deeper than 3.0 km. Meanwhile, with an increasing CO_2_ mole fraction in the gaseous phase from 0.1% to 10.0% in carbonate systems, the aqueous concentration of metal ions increases, e.g., dissolved amount of CaFe_0.7_Mg_0.3_(CO_3_)_2_ increases and reaches maximum of 1.78 mmol·L^-1^ and 8.26 mmol·L^-1^ at burial depth of 0.7 km with CO_2_ mole fraction of 0.1% and 10.0%, respectively. For the influence of overpressure in the calcite system, with overpressure ranging from 36 MPa to 83 MPa, pH reaches a minimum of 6.8 at overpressure of 51 MPa; meanwhile, Δ[Ca^2+^] increases slightly from -2.24 mmol·L^-1^ to -2.17 mmol·L^-1^ and remains negative, indicating it is also a precipitation process at burial depth of 3.9 km where overpressure generated. The method used in this study can be applied in assessing burial precipitation-dissolution processes and predicting possible pores in reservoirs with carbonate cement-water-carbon dioxide.

## Introduction

For petroleum reservoirs, about one-third are consisted of carbonate rocks, which include calcite and binary or ternary carbonates with Mg, Fe or Mn, e.g., dolomite (CaMg(CO_3_)_2_), ferrocalcite (Ca_0.9_Fe_0.1_CO_3_) and ankerite (CaFe_0.5_Mn_0.5_(CO_3_)_2_) [1,2]. Within these carbonate reservoirs, secondary pores are considered as the main storage space, and was formed via water-rock interaction at different burial depths during diagenesis [3–5]. Besides, for diagenesis of source rocks, organic acid and CO_2_ can be released into sandstone pores and may lead to the dissolution of carbonate and formation of secondary pores [6]. And in recent years, to cut CO_2_ emission into the atmosphere from fossil-fuel power stations, geological sequestration or underground storage of CO_2_ in depleted oil and gas reservoirs has been investigated [7–10]; as a result, the initial physic-chemical equilibrium between the fluid and carbonate cements can be disturbed and dissolution or precipitation of carbonate cements may occur accordingly [11]. Prediction of precipitation-dissolution of carbonates can be also applied in microbially induced calcium-carbonate precipitation(MICP), in which nucleation sites and enzyme of urease and carbonic anhydrate play important roles for this biochemical process, while factors of the calcium concentration (Ca^2+^), the concentration of dissolved inorganic carbon and the pH are critical for formation of carbonates [12–14].

To predict precipitation/dissolution of carbonate cements at different burial depth with CO_2_, systems of calcite or dolomite-water-carbon dioxide under acidic condition were typically analyzed via ion equilibrium as well as minimization of Gibbs free energy (Δ*G*) and equilibrium constant [15,16]. For example, for the carbonate cements, Δ[Ca^2+^], the variation of [Ca^2+^] for every 100 m of burial depths, was analyzed in calcite and dolomite system [3,16]: if Δ[Ca^2+^] is positive, dissolution of carbonate happens; otherwise, precipitation takes place.

During diagenesis of carbonate cements, the main factors concerned include temperature, pressure, pH, $P_{{\text{CO}}_{2}}$, variable rock composition, hydrologic regime, fluid composition, organic acid anion, etc. [17,18]. Among these factors, temperatures play an important role: For the rock-H_2_O-CO_2_ system at different temperatures of 55°C, 70°C and 100°C, the corrosion of feldspars, silica and clay minerals intensifies with increasing temperature [19]. For CO_2_ in these systems, aqueous concentration of metal ions increased with increasing $P_{{\text{CO}}_{2}}$ in carbonate cements [20,21].

Within carbonates, there are binary or ternary minerals of carbonate cements with Mg, Fe or Mn, which are common in reservoir of sandstones [1]; these carbonates are important in formation of secondary pores via precipitation/dissolution within systems of carbonates-H_2_O-CO_2_ in reservoirs. However, study on thermodynamic equilibrium model and ionic concentrations in these binary or ternary minerals has been few reported. Meanwhile, effect of variable rock composition in ankerite, e.g., CaFe_x_Mg_1-x_(CO_3_)_2_ (0≤x≤1) during diagenesis is also a concern in thermodynamic calculation because of unavailability of thermodynamic data.

Overpressure, another factor that influences thermodynamic equilibrium as well as dissolution/precipitation, can be found at reservoirs where fluid pressure exceeds the hydrostatic pressure [22]. The reason of overpressure can be attributed to disequilibrium compaction, diagenesis and hydrocarbon generation, which involves in total organic carbon and hydrogen index [22]. Overpressure generated in source rock can be estimated via parameters of hydrogen index and total organic carbon [23], and can also be used to analyze dissolution/precipitation of carbonate cements in source rocks.

In this work, the authors 1) calculated Δ*G* and equilibrium constant of reactions in carbonate cements-water-carbon dioxide systems via chemical thermodynamic principles, 2) used Δ[M^2+^] (M = Ca^2+^, Fe^2+^, Mg^2+^, or Mn^2+^) to predict precipitation/dissolution in systems of carbonate cements-water-carbon dioxide, and 3) discussed the parameters, e.g., temperature, pressure, depth, pH, $P_{{\text{CO}}_{2}}$ and overpressure, that influence the chemical equilibrium and precipitation/dissolution. This model can be applied in carbonate cements with binary or ternary minerals in ferrocalcite and ankerite (CaFe_x_Mg_1-x_(CO_3_)_2_, 0≤x≤1) systems for prediction of precipitation/dissolution in sandstone reservoirs.

## Method

### The thermodynamic equilibrium model

Temperature and pressure are important parameters in diagenesis at different burial depth; a temperature gradient of 0.03 K/m and a pressure gradient of 1.0 MPa/100m with a surface pressure of 10^5^ Pa are given in this work.

The main reactions and equilibrium constant expressions in water-carbon dioxide system and carbonate cement dissociations are listed in Table 1.

**Table 1:** The equilibrium reactions and equilibrium constant expressions in carbonate-H_2_O-CO_2_ system

Equilibrium reactions in carbonate-H_2_O-CO_2_	Equilibrium constant expressions^-a^
CO_2_(g) + H_2_O ⇔ H_2_CO_3_	${\text{K}}_{1}=\frac{\left[{\text{H}}_{2}{\text{CO}}_{3}\right]}{{[\text{P}}_{{\text{CO}}_{2}}]}$
${\text{H}}_{2}{\text{CO}}_{3}\Leftrightarrow {\text{H}}^{+}+{\text{HCO}}_{3}^{-}$	${\text{K}}_{2}=\frac{\left[{\text{H}}^{+}{][\text{HCO}}_{3}^{-}\right]}{{[\text{H}}_{2}{\text{CO}}_{3}]}$
${\text{HCO}}_{3}^{-}\Leftrightarrow {\text{H}}^{+}+{\text{CO}}_{3}{}^{2-}$	${\text{K}}_{3}=\frac{\left[{\text{H}}^{+}{][\text{CO}}_{3}^{2-}\right]}{{[\text{HCO}}_{3}{}^{-}]}$
H_2_O ⇔ H^+^ + OH^−^	K_4_ = [H^+^][OH^−^]
${\text{CaCO}}_{3}\Leftrightarrow {\text{Ca}}^{2+}+{\text{CO}}_{3}^{2-}$	${\text{K}}_{5}=[{\text{Ca}}^{2+}{][\text{CO}}_{3}^{2-}]$
${\text{CaMg}(\text{CO}}_{3}{)}_{2}\Leftrightarrow {\text{Ca}}^{2+}+{\text{Mg}}^{2+}+{2\text{CO}}_{3}^{2-}$	${\text{K}}_{6}={[\text{Ca}}^{2+}{][\text{Mg}}^{2+}{][\text{CO}}_{3}^{2-}{]}^{2}$
${\text{Ca}}_{0.9}{\text{Fe}}_{0.1}{\text{CO}}_{3}\Leftrightarrow {0.9\text{Ca}}^{2+}+{0.1\text{Fe}}^{2+}+{\text{CO}}_{3}^{2-}$	${\text{K}}_{7}={{[\text{Ca}}^{2+}]}^{0.9}{{[\text{Fe}}^{2+}]}^{0.1}{[\text{CO}}_{3}^{2-}]$
$\begin{array}{l} {\text{CaFe}}_{0.5}{\text{Mg}}_{0.5}{\left({\text{CO}}_{3}\right)}_{2}\Leftrightarrow {\text{Ca}}^{2+}+0.5{\text{Mg}}^{2+}\\ +{0.5\text{Fe}}^{2+}+{2\text{CO}}_{3}^{2-} \end{array}$	${\text{K}}_{8}={[\text{Ca}}^{2+}{][\text{Mg}}^{2+}{]}^{0.5}{{[\text{Fe}}^{2+}]}^{0.5}{{[\text{CO}}_{3}^{2-}]}^{2}$
$\text{CaFe}({\text{CO}}_{3}{)}_{2}\Leftrightarrow {\text{Ca}}^{2+}+{\text{Fe}}^{2+}+{2\text{CO}}_{3}^{2-}$	${\text{K}}_{9}={[\text{Ca}}^{2+}{][\text{Fe}}^{2+}{][\text{CO}}_{3}^{2-}{]}^{2}$
${\text{MgCO}}_{3}\Leftrightarrow {\text{Mg}}^{2+}+{\text{CO}}_{3}^{2-}$	${\text{K}}_{10}={[\text{Mg}}^{2+}{][\text{CO}}_{3}^{2-}]$
${\text{FeCO}}_{3}\Leftrightarrow {\text{Fe}}^{2+}+{\text{CO}}_{3}^{2-}$	${\text{K}}_{11}={[\text{Fe}}^{2+}{][\text{CO}}_{3}^{2-}]$
$\begin{array}{l} {\text{CaFe}}_{0.5}{\text{Mn}}_{0.5}{\left({\text{CO}}_{3}\right)}_{2}\Leftrightarrow {\text{Ca}}^{2+}+{0.5\text{Fe}}^{2+}\\ +{0.5\text{Mn}}^{2+}+{2\text{CO}}_{3}^{2-} \end{array}$	${\text{K}}_{12}={[\text{Ca}}^{2+}{][\text{Mn}}^{2+}{]}^{0.5}{{[\text{Fe}}^{2+}]}^{0.5}{{[\text{CO}}_{3}^{2-}]}^{2}$
${\text{MnCO}}_{3}\Leftrightarrow {\text{Mn}}^{2+}+{\text{CO}}_{3}^{2-}$	${\text{K}}_{13}={[\text{Mn}}^{2+}{][\text{CO}}_{3}^{2-}]$

^-a^ [X] in mmol·L^-1^ represents the concentration of species X, and K is the thermodynamic equilibrium constant

In the current work, the concentrations of carbonate were used because in dilute solutions, concentrations of carbonates are approximately equal to the activities, and the calculation process can be simplified [16].

The direction of those reactions can be analyzed by the Gibbs free energy (Δ*G*), while Δ*G* at different temperatures and pressures can be calculated via Eq 1 [24]:

$$\Delta_{\text{r}}G^{0}=\Delta_{\text{r}}H_{T_{\text{ref}}}^{0}-T\Delta_{\text{r}}S{}_{{\text{T}}_{\text{ref}}}^{0}+\int_{T_{\text{ref}}}^{T}\Delta_{\text{r}}C_{P}\text{d}T-\int_{T_{\text{ref}}}^{T}\frac{\Delta_{\text{r}}C_{P}}{T}\text{d}T+\int_{P_{\text{ref}}}^{P}\Delta_{\text{r}}V\text{d}P$$ (1)

where:

$\Delta_{\text{r}}H_{T_{\text{ref}}}^{0}$ standard molar enthalpy of the reaction at 298.15 K and 10^5^ Pa

$\Delta_{\text{r}}S_{T_{\text{ref}}}^{0}$ standard molar entropy of the reaction at 298.15 K and 10^5^ Pa

*C*_*P*_ heat capacity at constant pressure, *C*_*P*_/J·K^−1^·mol^−1^ = *a* + *b*(*T*/K) + *c*(*T*/K)^−2^, in which *a*, *b* and *c* are the coefficients in the heat capacity polynomial.

*V* molar volume of solid phase and liquid phase

*T*_ref_ the temperature at 298.15K

*P*_ref_ the pressure at 10^5^ Pa

$$\int_{10^{5}}^{P}\Delta_{\text{r}}V\text{d}P=[(\sum {}_{\text{B}}v_{\text{B}}V_{\text{S}}^{0}{)}_{\text{production}}-{\left(\sum {}_{\text{B}}v_{\text{B}}V_{\text{S}}^{0}\right)}_{\text{reactant}}](P-10^{5})+v_{\text{B}}\int_{10^{5}}^{P}\Delta_{\text{r}}V_{\text{m}}\text{d}P$$ (2)

where:

*V*_s_ the molar volume of solid phase, including ion and solid mineral phase.

*V*_m_ the molar volume of liquid phase, which was calculated by the Soave-Redlich-Kwong (SRK) equation, which is capable to predict molar volume of CO_2_ and H_2_O, as listed in Eq 3 [25,26].

$$P=\frac{RT}{V-b}-\frac{a\alpha (T)}{V(V+b)}$$ (3)

where

$$a=0.42748\frac{R^{2}T_{c}^{2}}{P_{c}}$$ (4)

$$b=0.08664\frac{RT_{c}^{}}{P_{c}}$$ (5)

$$\alpha (T)={\left(1+(0.485+1.574\omega -0.176\omega^{2})(1-\sqrt{T_{r}})\right)}^{2}$$ (6)

The critical temperature (*T*_c_), critical pressure (*P*_c_) and acentric factor (*ω*) were applied in SRK equation and are listed in Table 2 [27].

**Table 2:** The critical values of carbon dioxide and water

	*T*_c_(K)	*P*_*c*_(bar)	*ω*
CO_2_(g)	304.2	73.8	0.224
H_2_O	647.1	220.6	0.345

Based on Δ*G*, the equilibrium constant of reactions (K) can be calculated via Eq 7:

$$\Delta G=\Delta G_{0}+RT\text{lnK}$$ (7)

in which Δ*G*_0_ is the standard Gibbs free energy of the reaction at 298.15K and 10^5^ Pa, *T* is the absolute temperature in Kelvin, and *R* is the gas constant (8.314 J·K^−1^·mol^−1^).

In the system of calcite-water-carbon dioxide, the charge balance equations or electroneutrality are calculated by the positive and negative ions as followed [28]:

$${[\text{H}}^{+}]+{2[\text{Ca}}^{2+}]={2[\text{CO}}_{3}^{2-}]+{[\text{HCO}}_{3}^{-}]+{[\text{OH}}^{-}]$$ (8)

For the systems of the dolomite or ankerite, Eq 8 can be revised as Eq 9.

$${[\text{H}}^{+}]+{2[\text{Ca}}^{2+}]+{2[\text{Fe}}^{2+}]+{2[\text{Mg}}^{2+}{]\;(\text{or}\;[\text{Mn}}^{2+}])={2[\text{CO}}_{3}^{2-}]+{[\text{HCO}}_{3}^{-}]+{[\text{OH}}^{-}]$$ (9)

According to the equilibrium expressions of H_2_O-CO_2_ (Table 1), electroneutrality equation (Eq 9) with different carbon dioxide pressure ($P_{{\text{CO}}_{2}}$) and the equilibrium constants (K or K^’^) of carbonate, the quartic equation related to [H^+^] can be obtained via Eqs 10 and 11, as followed:

$$(\frac{2\text{K}}{{\text{K}}_{1}\cdot {\text{K}}_{2}\cdot {\text{K}}_{3}\cdot (P_{{\text{CO}}_{2}})}{)[\text{H}}^{+}{]}^{4}+{{[\text{H}}^{+}]}^{3}-({\text{K}}_{1}\cdot {\text{K}}_{2}\cdot (P_{{\text{CO}}_{2}})+{\text{K}}_{4}{)[\text{H}}^{+}]-2(P_{{\text{CO}}_{2}})\cdot {\text{K}}_{1}\cdot {\text{K}}_{2}\cdot {\text{K}}_{3}=0$$ (10)

$$(\frac{4\sqrt{K^{\prime }}}{K_{1}\cdot K_{2}\cdot K_{3}\cdot (P_{CO_{2}})}){\left[H^{+}\right]}^{4}+{\left[H^{+}\right]}^{3}-(K_{1}\cdot K_{2}\cdot (P_{CO_{2}})+K_{4})[H^{+}]-2(P_{CO_{2}})\cdot K_{1}\cdot K_{2}\cdot K_{3}=0$$ (11)

Eq 10 can be applied to in the systems of MCO_3_−CO_2_−H_2_O (M = Ca, Fe, Mg or Mn) and Ca_0.9_Fe_0.1_CO_3_−CO_2_−H_2_O, while Eq 11 can be applied to in the system of dolomite or ankerite.

Based on [H^+^], the concentration of other species, such as [Ca^2+^], [${\text{HCO}}_{3}^{-}$] and [OH^−^], can be calculated according to equilibrium expressions and equilibrium constant of reaction, while Δ[Ca^2+^], the variation of [Ca^2+^] equilibrium concentration for every 100 m of burial depths, can be calculated to predict precipitation or dissolution occurring in carbonate cement system.

### Thermodynamic data of ferrocalcite and ankerite

The standard Gibbs free energy of formation (Δ_f_*G*_0_) of minerals can be found from databases or literatures [29–31], while neither equilibrium constant nor Δ_f_*G*_0_ for ferrocalcite (Ca_0.9_Fe_0.1_CO_3_) and ankerite (CaFe_0.5_Mg_0.5_(CO_3_)_2_) can be found to calculate the equilibrium concentration. However, a linear correlation exists between Gibbs free energies of formation of calcite-like carbonate and the corresponding aqueous divalent cations, and can be used to estimate the basic thermodynamic value of ferrocalcite (Ca_0.9_Fe_0.1_CO_3_) [32]. Meanwhile, the equation of ankerite dissociation (Eq 12) can be deduced by combining calcite dissociation and Eq 13, and the corresponding equilibrium constant of ankerite dissociation (CaFe_0.5_Mg_0.5_(CO_3_)_2_), K_8_, is shown in Eq 14, while K^*^ is the equilibrium constant of Eq 13:

$${\text{CaFe}}_{0.5}{\text{Mg}}_{0.5}{{(\text{CO}}_{3})}_{2}\Leftrightarrow {\text{Ca}}^{2+}+{0.5\text{Fe}}^{2+}+{0.5\text{Mg}}^{2+}+{2\text{CO}}_{3}^{2-}$$ (12)

$${2\text{CaCO}}_{3}+{0.5\text{Fe}}^{2+}+{0.5\text{Mg}}^{2+}\Leftrightarrow {\text{Ca}}^{2+}+{\text{CaFe}}_{0.5}{\text{Mg}}_{0.5}{{(\text{CO}}_{3})}_{2}$$ (13)

$${\text{K}}_{8}={[\text{Ca}}^{2+}{][\text{Fe}}^{2+}{]}^{0.5}{{[\text{Mg}}^{2+}]}^{0.5}{{[\text{CO}}_{3}^{2-}]}^{2}=\frac{{\text{K}}_{1}^{2}}{{\text{K}}^{*}}$$ (14)

$${\text{K}}^{*}=\frac{{[\text{Ca}}^{2+}]}{{{[\text{Fe}}^{2+}]}^{0.5}{{[\text{Mg}}^{2+}]}^{0.5}}=\frac{{[\text{Ca}}^{2+}{][\text{CO}}_{3}^{2-}]}{{{[\text{Fe}}^{2+}]}^{0.5}{{[\text{Mg}}^{2+}]}^{0.5}{[\text{CO}}_{3}^{2-}]}$$ (15)

The thermodynamic data used in this work are shown in Table 3.

**Table 3:** The thermodynamic data of carbonate minerals. Data from [33] and [34] are marked as−^a^ and−^b^.

Species	Δ_f_*H* (kJ/mol)	*S* (J/mol/K)	*V* (cm^3^/mol)	*C*_*p*_ / J·K^−1^·mol^−1^ = *a* + *b*(*T* / K) + *c*(*T* / K)^−2^		
				*a*	*b*	*c*
Calcite^-a^	-1207.88	92.50	36.89	140.9	0.005029	-950700
Dolomite^-a^	-2325.76	156.10	64.29	358.9	-0.00495	0
Magnesite^-a^	-1110.93	65.50	28.03	186.4	-0.003772	0
Siderite^-a^	-762.22	93.30	29.43	168.4	0	0
Rhodochrosite^-a^	-892.28	98.00	31.07	169.5	0	0
CaFe(CO_3_)_2_^-a^	-1970.62	188.46	66.06	341.0	0.001161	0
Ca_0.1_Fe_0.9_(CO_3_)_2_	-1178.19	92.678	36.16	87.1	0.052325	2093000
CO_2_(g)^-a^	-393.51	213.7	0	87.8	-0.002644	706400
H_2_O(aq)^-b^	-285.8	70	18.07	30.5	0.0103	0
H_2_CO_3_^-b^	-699.648	187.443	-	0.000	0.70291	0
Ca^2+-a^	-543.3	-56.50	-18.06	0	0.069	0
Mg^2+-a^	-465.96	-138.10	-21.55	0	0.0462	0
Fe^2+-a^	-90.42	-107.11	-22.20	0	0	0
Mn^2+-a^	-220.39	-73.57	-17.1	0	0.04184	0
H^+-a^	0	0	0	0	0	0
OH^−-a^	-230.02	-10.71	-4.18	0	0	0
HCO_3_^−-b^	-691.992	91.211	24.6	0.000	-0.12468	0
${\text{CO}}_{3}^{2-}$^-a^	-675.23	-50.00	-5.02	0	0	0

Because of the instability in solution, carbonic acid can be treated as CO_2_(g) dissolved in water as CO_2_(aq) [35,36]. When $\frac{T}{T_{\text{c}}}$≤0.98, Bhirud Equation in Eq 16 can be applied to calculate its molar volume; when $\frac{T}{T_{\text{c}}}$>0.98, molar volume is considered as a constant because the influence of temperature and pressure on molar volume is slight in the liquid phase [37].

$$\ln(\frac{P_{c}V}{RT})=\ln u^{0}+\ln u^{1}=\ln u$$ (16)

Where: *u*^0^ and *u*^1^ are dimensionless and are a function of $T_{r}=\frac{T}{T_{\text{c}}}$.

$$\begin{array}{l} \ln u^{0}=-0.40062-8.0006T_{\text{r}}+49.3780T_{\text{r}}^{2}-170.6616T_{\text{r}}^{3}+287.6989T_{\text{r}}^{4}\\ -232.5608T_{\text{r}}^{5}+73.03299T_{\text{r}}^{6} \end{array}$$ (17)

$$\begin{array}{l} \ln u^{1}=13.4412-135.7437T_{\text{r}}+533.38T_{\text{r}}^{2}-1091.453T_{\text{r}}^{3}+1231.43T_{\text{r}}^{4}\\ -728.227T_{\text{r}}^{5}+176.737T_{\text{r}}^{6} \end{array}$$ (18)

To verify the estimated thermodynamic data obtained by the current thermodynamic calculation method (TCM), the Helgeson-Kirkham-Flowers (HKF) equation was used for comparison [38]. The equilibrium constants of bicarbonate (K_3_) and water dissociation (K_4_) were selected and calculated by methods of TCM and HKF, respectively, and the results and relative differences are listed in Table 4; the results indicate that TCM is an effective way to calculate the equilibrium constant, as compared with HKF equation, while less parameters are needed for TCM.

**Table 4:** Comparison of equilibrium constants calculated by thermodynamic calculation method (TCM) and HKF equation in system of CO_2_-H_2_O

*P*(Pa)	*T*(K)	K_3_			K_4_		
		TCM	HKF^-a^	△	TCM	HKF^-a^	△
1.0	298.15	4.57×10^−11^	4.69×10^−11^	-2.56%	1.03×10^−14^	1.00×10^−14^	2.91%
1000.0	373.15	2.38×10^−10^	2.42×10^−10^	-1.65%	5.48×10^−13^	5.38×10^−13^	1.86%

^-a^ [3]

### The effect of variable composition in ankerite system and overpressure in carbonate systems

The thermodynamic method was used to analyze the influence of variable rock composition in the ankerite system (CaFe_x_Mg_1-x_(CO_3_)_2_, 0≤x≤1), in which CaFe_0.2_Mg_0.8_(CO_3_)_2_, CaFe_0.3_Mg_0.7_(CO_3_)_2_, CaFe_0.5_Mg_0.5_(CO_3_)_2_, CaFe_0.7_Mg_0.3_(CO_3_)_2_ and CaFe(CO_3_)_2_ are selected and named as ank2, ank3, ank5, ank7 and ank10, respectively.

Overpressure is defined as fluid pressure exceeding the hydrostatic pressure, and is always found in the burial depth from 3.2 km and 3.9 km; for example, overpressure was reported with variation up to 83 MPa at 3.9 km in smulation experiments [23]. Based on the values of overpressure from 36 MPa to 83 MPa, a CaFe(CO_3_)_2_ system with 800 mg/g of hydrogen index at a burial depth of 3.9 km was selected, and data of Δ_r_*G* and equilibrium constant were calculated, as listed in Table 5.

**Table 5:** The overpressure, Δ_r_*G* and equilibrium constant in CaFe(CO_3_)_2_- H_2_O- CO_2_ system

Overpressure(MPa)	Δ_r_*V*^0^(*P*−*P*^0^)(m^3^·Pa^−1^·mol^−1^)	Δ_*r*_*G*(kJ·mol^−1^)	lgK
36	-4177.32	176.44	-22.20
51	-5922.72	174.70	-21.98
65	-7551.76	173.07	-21.78
78	-9064.44	171.56	-21.59
83	-9646.24	170.97	-21.51

in which *P*^0^ is the pressure at 10^5^ Pa

**Fig 1:**
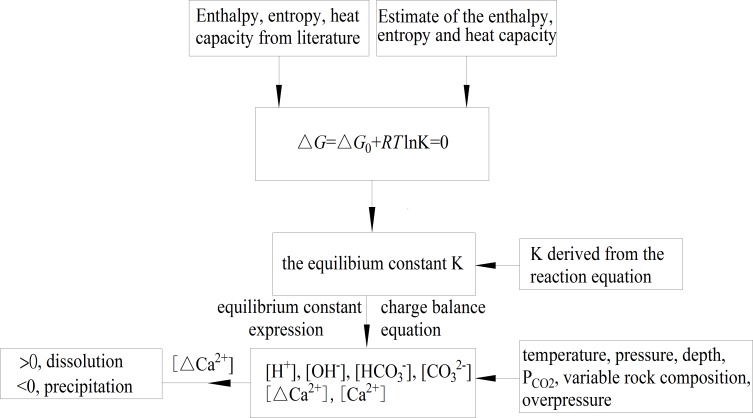
Flow diagram of the calculation method.

The overall methodology is shown in Fig 1: the minimization of Δ*G* and equilibrium constant of reactions were calculated in carbonate cements-water-carbon dioxide systems via chemical thermodynamic parameters, and the Δ[M^2+^] (M = Ca^2+^, Fe^2+^, Mg^2+^, or Mn^2+^) were analyzed to predict precipitation/dissolution with variable parameters, e.g., temperature, pressure, depth, pH, $P_{{\text{CO}}_{2}}$, variable rock composition and overpressure.

## Results and Discussion

### Chemical equilibrium constant of reactions in carbonate-water-carbon dioxide system

The equilibrium constants calculated from Δ*G* of reaction can be used to calculate equilibrium constant and to analyze equilibrium concentration of ions, such as [Ca^2+^], [Mg^2+^], [Fe^2+^] or [Mn^2+^]. The influence of 1) temperature, 2) pressure and 3) burial depth on equilibrium constant of calcite-H_2_O-CO_2_ was firstly analyzed.

As shown in Table 6, when the temperature increases from 301.15 K to 478.15 K, lgK decreases from -8.53 to -9.69; meanwhile, when the pressure increases from 15 to 70 MPa at 301.15 K, lgK increase slightly from -8.53 to -7.82. The result indicates that temperature has a higher impact on lgK on an opposite direction, as compared with pressure. As a result, when the burial depth increases from 1.5 km to 6.0 km, the temperature increases from 343.15 K to 478.15K and pressure increases from 15 MPa to 60 MPa; meanwhile, lgK decreases from -8.69 to -9.39.

**Table 6:** Δ*G* and equilibrium constants (K) with different temperatures, pressures and burial depths in calcite-H_2_O-CO_2_

Depth(km)	Temperature(K)	Pressure(MPa)	Δ*G*(J/mol)	lgK
-	301.15	15	49166.56	-8.53
-	478.15	15	88688.97	-9.69
-	301.15	50	46288.02	-8.03
-	301.15	70	45088.62	-7.82
0	298.15	0.1	48681.85	-8.53
1.5	343.15	15	57103.05	-8.69
4.0	418.15	40	72544.71	-9.06
6.0	478.15	60	85970.32	-9.39

**Fig 2:**
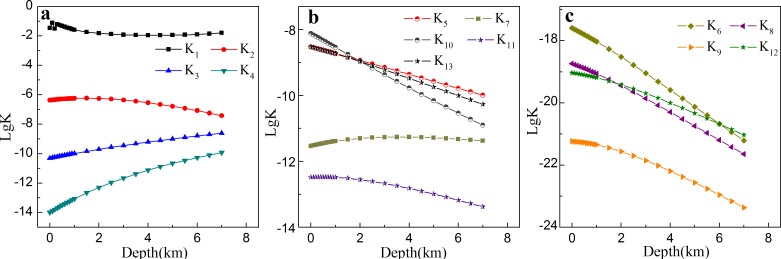
The equilibrium constants (K_1_-K_13_) at different burial depth in carbonate-water-carbon dioxide system. (a): the equilibrium constants in water-carbon dioxide systems; (b-c): the equilibrium constants in carbonate cements-water-carbon dioxide.

Based on the aforementioned method, the equilibrium constants of carbonates-H_2_O-CO_2_ at different depths were then calculated, as shown in Fig 2. The results indicate that lgK are negative at different burial depth, while the Δ*G* are positive (Δ*G* = −*RT* ln K), indicating that those processes cannot take place automatically.

For the water-carbon dioxide system in Fig 2A, the equilibrium constant of carbon dioxide dissolving in water and forming carbonic acid, lgK_1_, ranges from -1.12 to -1.97 and reaches the minimum of -1.97 at depth of 4.0 km. LgK_2_ (the equilibrium constant of carbonic acid dissociation) reaches the maximum of -6.23 at about 1.5 km, and then decreases continuously with further increasing depth. The equilibrium constant of secondary dissociation of carbonic acid, lgK_3_, ranges from -10.31 to -8.60 at the depth from 0.0 km to 7.0 km, which is about three orders of magnitude lower than K_2_. Furthermore, the equilibrium constant of water dissociation, lgK_4_, is increasing from -14.00 to -10.10 with increasing burial depth.

For the equilibrium constant of carbonate cement dissociation in Fig 2B, calcite (K_5_) and magnesite (K_10_) show a similar trend of decreasing with burial depth increased. Meanwhile, the equilibrium constant of ferrocalcite dissociation (K_7_) locates between those of calcite (K_5_) and siderite (K_11_); in the mean time, the equilibrium constants of dolomite (K_6_), CaFe_0.5_Mg_0.5_(CO_3_)_2_ (K_8_), CaFe(CO_3_)_2_ (K_9_) and CaFe_0.5_Mn_0.5_(CO_3_)_2_ (K_12_) in Fig 2C are several orders of magnitude lower than those of other carbonate cements in Fig 2B. Through the equilibrium constants, the concentrations of dissolved species in aqueous solution were then calculated.

### Calculation of Δ[M^2+^] for prediction of precipitation/dissolution of carbonate cements with different CO_2_ mole fraction

The data of 1) Δ[Ca^2+^] in calcite, dolomite, ferrocalcite and ankerite, 2) Δ[Fe^2+^] in siderite, 3) Δ[Mg^2+^] in magnesite and 4) Δ[Mn^2+^] in rhodochrosite were analyzed; in these cases, Δ[M^2+^] is defined as the variation of equilibrium [M^2+^] for every 100 m of burial depths, and can indicate the dissolution or precipitation during diagenesis.

**Fig 3:**
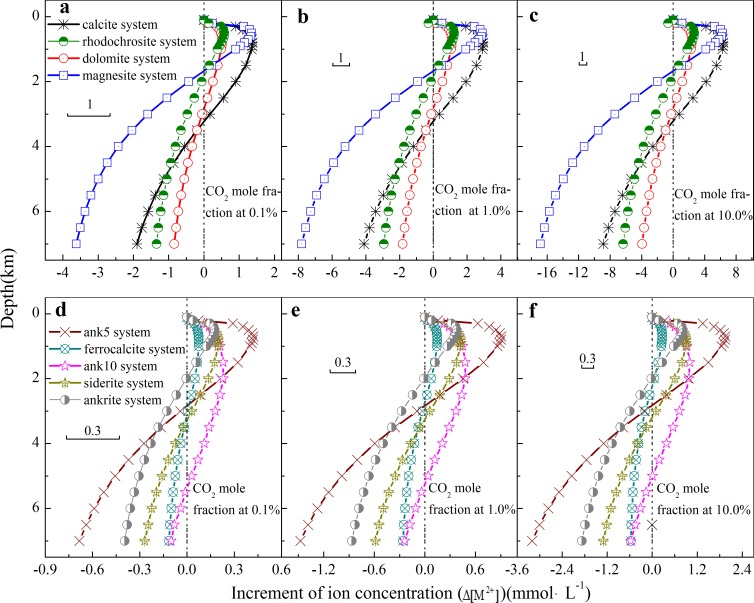
The increment of ion concentration, Δ[M^2+^], as a function of depth in carbonate cement systems with 1) Δ[Ca^2+^] in calcite, dolomite, ferrocalcite and ankerite, 2) Δ[Fe^2+^] in siderite, 3) Δ[Mg^2+^] in magnesite and 4) Δ[Mn^2+^] in rhodochrosite, in which Δ[M^2+^] is defined as the variation of [M2+] for every 100 m of burial depths.

As shown in Fig 3A, with the CO_2_ mole fraction at 0.1%, the systems of calcite, dolomite, magnesite and rhodochrosite are shown a trend of dissolving because Δ[M^2+^] are positive at depth from 0.0 km to 3.2 km, 2.7 km, 1.8 km and 2.0 km, respectively, indicating the secondary pores may form during this dissolution process; and in this dissolution process, the systems of calcite, dolomite, magnesite and rhodochrosite produce maximal concentrations of 1.37, 0.52, 1.35 and 0.58 mmol·L^-1^ at depth of 0.8 km, 0.7 km, 0.5 km and 0.5 km. As to systems of calcite and dolomite, the amount of dolomite dissolved was smaller than that of calcite at different depth, thus the dolomitization of limestone may decrease the porosity [39,40]. With further increasing depth deeper than 3.2 km, 2.7 km, 1.8 km and 2.0 km in cements of calcite, dolomite, magnesite and rhodochrosite, respectively, Δ[M^2+^] turns to negative, suggesting that precipitation occurs in these systems.

On the other hand, carbon dioxide partial pressure has effect on these systems of carbonate cements-water-carbon dioxide, as shown in Fig 3: with increasing CO_2_ mole fraction, the systems of calcite, dolomite, magnesite and rhodochrosite still show a similar trend of dissolving firstly and then precipitating with the increasing depth, while the maximal values are also obtained at the same depths. Nevertheless, the maxima of dissolving amount in systems of calcite, dolomite, magnesite and rhodochrosite were obtained near depth of 0.5–0.8 km, and increase by about four times with the partial pressure of carbon dioxide increasing from 0.1% to 10.0%, indicating that increasing partial pressure of CO_2_ results in the instability or dissolution of these carbonate cements, thus promoting the formation of secondary pore [41].

The relationship between Δ[M^2+^] and depth in cements of CaFe_0.5_Mg_0.5_(CO_3_)_2_, Ca_0.9_Fe_0.1_(CO_3_)_2_, CaFe(CO_3_)_2_, FeCO_3_ and CaFe_0.5_Mn_0.5_(CO_3_)_2_ at different CO_2_ mole fraction are shown in Fig 3D–3F. These five carbonate cements show a similar trend of dissolution firstly followed by precipitation. For example, in the CaFe_0.5_Mg_0.5_(CO_3_)_2_ system, dissociation happens within 0.0–3.0 km and precipitation occurs with depth deeper than 3.0 km, which is similar to the results reported by [1]: in Wilcox (Eocene) sandstones, ankerite (CaFe_0.5_Mg_0.5_(CO_3_)_2_) precipitates at depths deeper than 3.2 km. The slight difference in depth can be attributed to geological factors, such as the burial history, organic acid anions and chemical compaction. In Fig 3D–3F, with the carbon dioxide mole fraction increasing from 0.1% to 10.0%, the maximal amounts of dissolved cements increase and obtained near depth of 0.7–0.9 km. For example, the maximal dissolved siderite (FeCO_3_) at depth of 0.7 km increases from 0.21 mmol·L^-1^ to 0.86 mmol·L^-1^ with CO_2_ mole fraction increasing from 0.1% to 10.0%.

### The relationship between carbonate species concentration and $P_{{\text{CO}}_{2}}$ in ankerite (CaFe_0.5_Mg_0.5_ (CO_3_)_2_) system at depth of 2.5 km

**Fig 4:**
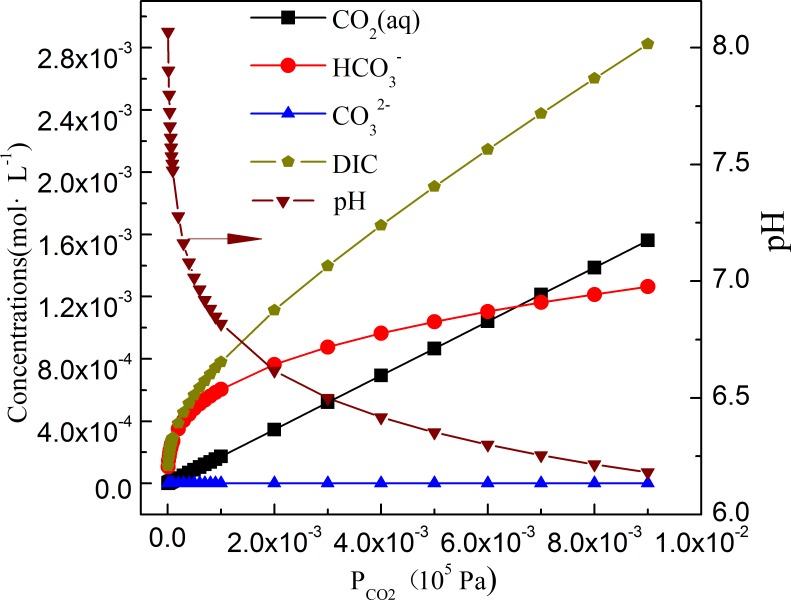
Distribution of carbonate species in CaFe_0.5_Mg_0.5_(CO_3_)_2_ system as a function of $P_{{\text{CO}}_{2}}$ at 373.15 K and 25 MPa. (DIC, total dissolved inorganic carbon, $\text{DIC}={[\text{CO}}_{2}(\text{aq})]+{[\text{HCO}}_{3}^{-}]+{[\text{CO}}_{3}^{2-}]$)

For carbonate cements-H_2_O-CO_2_ systems with different cements and CO_2_ mole fraction, carbonate species, e.g., HCO_3_^-^ and CO_3_^2-^, play an important role in dissolution/precipitation of cements. System of CaFe_0.5_Mg_0.5_(CO_3_)_2_ was then selected as a model to analyze effect of CO_2_ mole fraction on variation of carbonate species (total dissolved inorganic carbon, $\text{DIC}={[\text{CO}}_{2}(\text{aq})]+{[\text{HCO}}_{3}^{-}]+{[\text{CO}}_{3}^{2-}]$) at burial depth of 2.5 km with 373.15 K and 25 MPa. As shown in Fig 4, pH decrease from 7.6 to 6.2 with $P_{{\text{CO}}_{2}}$ increasing from 0 MPa to 1.0×10^−3^ MPa, while the total amount of DIC, [CO_2_(aq)], $\left[{\text{HCO}}_{3}^{-}\right]$ increase continuously. $\left[{\text{CO}}_{3}^{2-}\right]$ varies at a range of 1.28×10^−3^–1.95×10^−4^ mmol·L^-1^, and is about three orders of magnitude smaller than that of $\left[{\text{HCO}}_{3}^{-}\right]$.

### The pH at different depth in carbonate cements-H_2_O-CO_2_ systems

**Fig 5:**
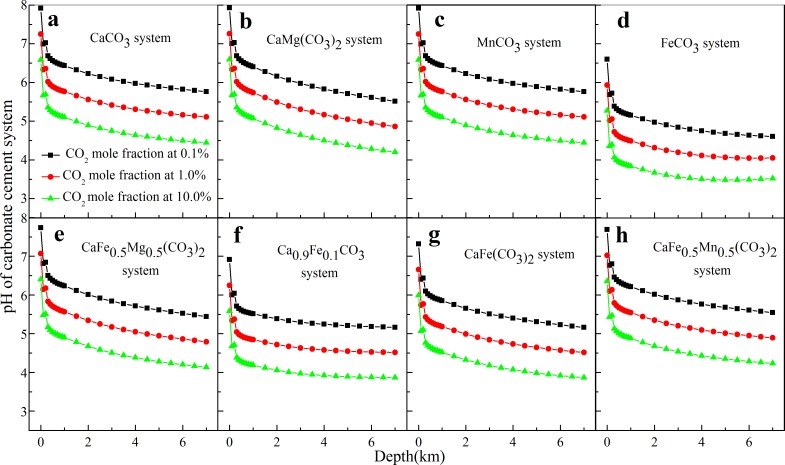
pH of carbonate cements-H_2_O-CO_2_ system with different burial depth and CO_2_ mole fraction.

The relationship between pH at different CO_2_ mole faction and depth in carbonate cement systems was also analyzed and is shown in Fig 5. For the calcite system in Fig 5A, the pH decreases from 7.9 to 5.8 with increasing burial depth from 0.0 km to 7.0 km at CO_2_ mole fraction 0.1%, and this variation can be attributed to the dissolution of primary minerals [42]. It is worth to note that as the depth increases from 0.0 km to 1.0 km, pH decreases sharply from 7.9 to 6.4. For the effect of CO_2_ mole fraction, pH decreases from 6.0 to 5.3 and to 4.6 with CO_2_ mole fraction increasing from 0.1% to 1.0% and to 10.0%, respectively, at depth of 4.0 km.

Other carbonate cements in Fig 5B–5H show a similar trend of decreasing pH with increasing CO_2_ mole fraction. Among these cements, the siderite-water-carbon dioxide system produces the lowest pH of 3.5 at depth of 4.0 km (CO_2_ mole fraction 10.0%), which can be attributed to hydrolysis of ferrous ion [43].

### The main dissolved species generated during the dissolution process in carbonate cements

**Fig 6:**
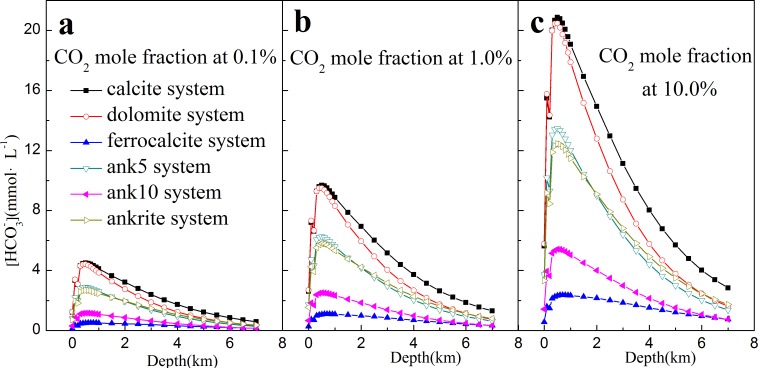
[HCO_3_^-^] as a function of depth with different CO_2_ mole fraction in carbonate cements-CO_2_-H_2_O systems.

To find the main species generated during the dissolution/precipitation process, the relationship between calcium and bicarbonate ion at different depth was analyzed. In Fig 6A of the calcite system, $\left[{\text{HCO}}_{3}^{-}\right]$ is increasing from 1.21 mmol·L^-1^ to a maximum of 4.32 mmol·L^-1^ as depth ranging from 0.0 km to 0.8 km with CO_2_ mole fraction of 0.1%; with depth deeper than 0.8 km, $\left[{\text{HCO}}_{3}^{-}\right]$ is decreasing to 0.59 mmol·L^-1^ at 7.0 km. Other carbonate cements also show a similar trend.

For the effect of CO_2_, with increasing CO_2_ mole fraction from 0.1% to 10.0%, $\left[{\text{HCO}}_{3}^{-}\right]$ increases remarkably at the same depth, as shown in Fig 6. For example, $\left[{\text{HCO}}_{3}^{-}\right]$ in the calcite system increases from 4.32 mmol·L^-1^ to 20.07 mmol·L^-1^ with CO_2_ mole fraction increasing from 0.1% to 10.0% at depth of 0.8 km.

**Fig 7:**
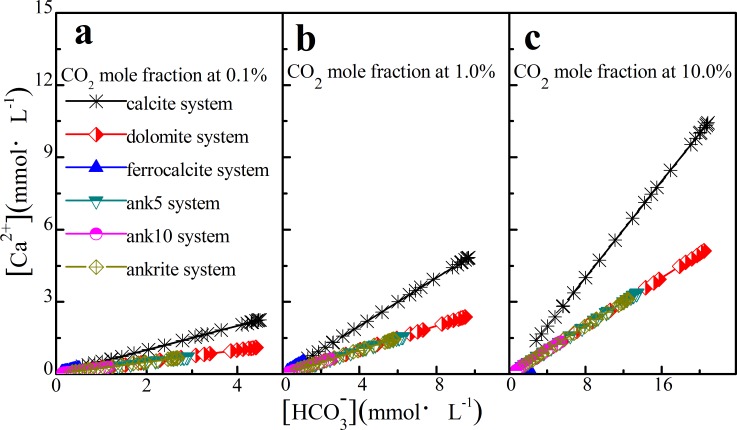
The linear relationship between [Ca^2+^] and [HCO_3_^-^].

The relationship between [Ca^2+^] and $\left[{\text{HCO}}_{3}^{-}\right]$ was studied as well, and a linear relationship was found, as shown in Fig 7: in the calcite system, $\left[{\text{HCO}}_{3}^{-}\right]$ increases from 1.21 mmol·L^-1^ to 4.32 mmol·L^-1^ at 0.1% of CO_2_ mole fraction when depth increasing from 0.0 km to 0.8 km, and [Ca^2+^] increases linearly from 0.61 mmol·L^-1^ to 2.16 mmol·L^-1^ (Fig 7A). With increasing CO_2_ mole fraction from 0.1% to 10.0%, there is still a linear relationship in other carbonate cements, as shown in Fig 7.

### The influence of variable rock composition in ankerite-H_2_O-CO_2_ system

**Fig 8:**
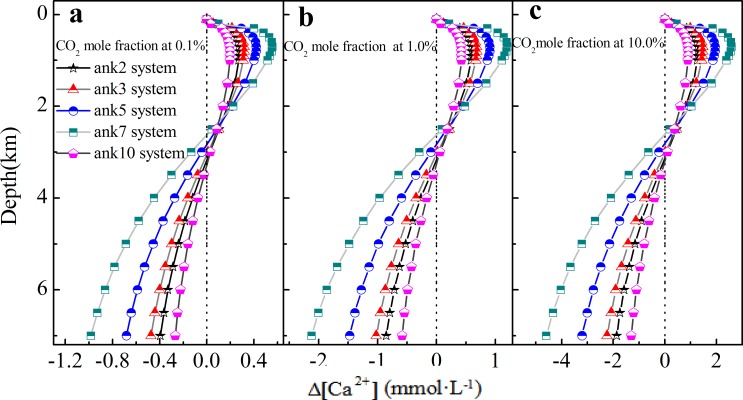
The variation of calcium ion at different burial depth in ankerite system with variable compositions.

Ankerite consists of variable compositions, and the influence of variable composition on precipitation/dissolution was analyzed. As shown in Fig 8A, Δ[Ca^2+^] indicates that ankerite (CaFe_x_Mg_1-x_(CO_3_)_2_, 0≤x≤1) system dissolves at depth smaller than 3.5 km with a maximal dissolved amount at depth of 0.8 km, but precipitates with depth deeper than 3.5 km. With the increasing Fe content (from ank2 to ank7), the amount of dissolved or precipitated carbonates is also increased. Meanwhile, CaFe(CO_3_)_2_ (ank10) is an exception: the amount of precipitation or dissolution is the lowest among these ankerite cements. The reason can be attributed to that the end-member of CaFe(CO_3_)_2_ mainly exists as a two-phase mineral with both calcite and siderite [44]. On the effect of CO_2_, Δ[Ca^2+^] increases by about four times with CO_2_ mole fraction increasing from 0.1% to 10.0%, which is shown in Fig 8, indicating that the amount of dissolved carbonate cements also increases with increasing CO_2_ mole fraction.

**Fig 9:**
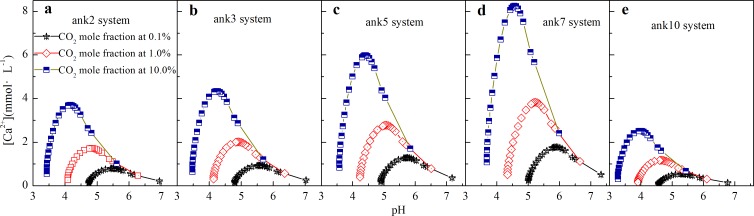
The relationship between pH and [Ca^2+^] in ankerite-H_2_O-CO_2_ system.

The relationship between pH and concentration of [Ca^2+^] in ankerite-H_2_O-CO_2_ system was also studied. As shown in Fig 9A of ank2 system, with the decrement of pH, [Ca^2+^] reaches the maximum of 0.80 mmol·L^-1^ at CO_2_ mole fraction of 0.1%, indicating dissolution may happen; then the [Ca^2+^] drops with pH further decreasing, indicating there can be precipitation and formation of carbonate cement. With increasing CO_2_ mole fraction, the maxima of [Ca^2+^] increase and emerge at lower pHs, e.g., 0.80 mmol·L^-1^ at pH = 5.5 for 0.1% of CO_2_, 1.72 mmol·L^-1^ at pH = 4.8 for 1.0% of CO_2_, and 3.70 mmol·L^-1^ at pH = 4.2 for 10.0% of CO_2_ in system of ank2. With increment of Fe content in ankerite (CaFe_x_Mg_1-x_(CO_3_)_2_, 0≤x≤1) system, as shown in Fig 9A–9D), the maximum of [Ca^2+^] also increases as well. The ank10 (CaFe(CO_3_)_2_) is an exception: as shown in Fig 9E, [Ca^2+^] varies from 0.3 mmol·L^-1^ to 2.5 mmol·L^-1^ in CaFe(CO_3_)_2_ at CO_2_ mole fraction of 10.0%, which is the lowest in ankerite system, and can also be attributed to that the end-member of CaFe(CO_3_)_2_ does not exist as one-phase mineral [44].

### The influence of overpressure on carbonate cements-H_2_O-CO_2_

**Fig 10:**
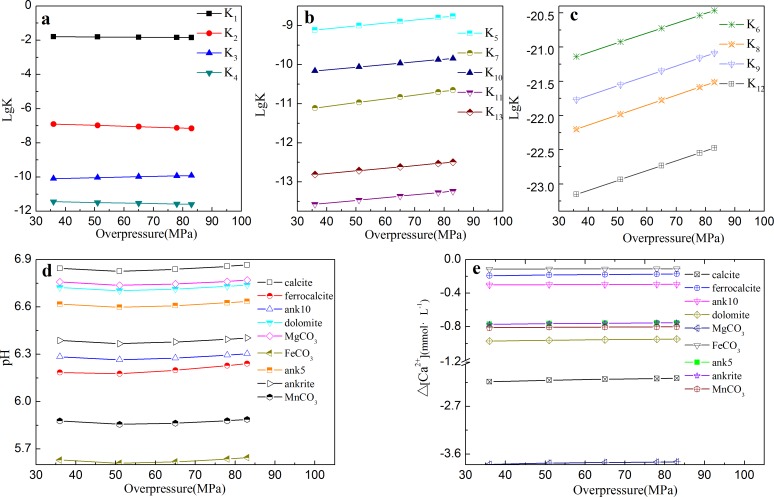
The equilibrium constant and overpressure at depth of 3.9 km with 800 mg/g of hydrogen index, (d) The relationship between overpressure and pH with CO_2_ mole fraction 0.1% at a burial depth of 3.9 km with 800 mg/g of hydrogen index, and (e) Δ[Ca^2+^] at different overpressures with CO_2_ mole fraction of 0.1%.

The pressure variation during diagenesis can be applied to estimate the overpressure and to calculate the equilibrium constant. Effect of overpressure on carbonates systems was studied at depth of 3.9 km with 800 mg/g of hydrogen index where overpressure generated. As shown in Fig 10A, with the increasing overpressure, the equilibrium constants of 1) formation of carbonic acid (K_1_), 2) formation of bicarbonate (K_2_) and 3) water dissociation (K_4_) decrease. Meanwhile, the equilibrium constants of dissociation of carbonate cements (K_5_-K_13_) increase with increasing overpressure in Fig 10B and 10C. For example, over ferrocalcite (Ca_0.9_Fe_0.1_CO_3_), the equilibrium constant (K_7_) ranges from 7.8×10^−12^ to 2.2×10^−11^ with overpressure increasing from 36 MPa to 83 MPa. The equilibrium constants of dolomite (K_6_), CaFe_0.5_Mg_0.5_(CO_3_)_2_ (K_8_) and CaFe(CO_3_)_2_ (K_9_) are several orders of magnitude lower than those of other carbonate cements.

The relationship between overpressure and pH was also analyzed, as shown in Fig 10D. The overpressure ranging from 36 MPa to 83 MPa at CO_2_ mole fraction of 0.1% leads to a minimal pHs of 6.8, 6.2, 6.3 and 6.4 at 51 MPa in the systems of calcite, ferrocalcite (Ca_0.9_Fe_0.1_CO_3_), ank10(CaFe(CO_3_)_2_) and ankerite(CaFe_0.5_Mn_0.5_(CO_3_)_2_), respectively. Meanwhile, system of siderite (FeCO_3_) produces the lowest pH at this range of overpressure.

Effect of overpressure on Δ[Ca^2+^] was studied as well. As shown in Fig 10E, in the calcite system, with increasing overpressure from 36 MPa to 83 MPa, Δ[Ca^2+^] increases slightly from -2.24 mmol·L^-1^ to -2.17 mmol·L^-1^ and remains negative, indicating it is still a precipitation process at depth of 3.9 km where overpressure generated. Other carbonate cements also show a similar trend of precipitation at depth of 3.9 km where overpressure generated, and the magnesite system produces the lowest Δ[Ca^2+^] from -3.79 mmol·L^-1^ to -3.75 mmol·L^-1^. This result is helpful to predict the accumulated space or reservoir formed in sandstone and source rock during diagenesis.

## Conclusions

The minimization of Δ*G* and equilibrium constant of reactions in carbonate cements-water-carbon dioxide systems were calculated via chemical thermodynamic principles. Δ[M^2+^], calculated from equilibrium concentration, was applied to predict the precipitation/dissolution process.

The results indicate that with increasing burial depth, carbonate cements with binary or ternary minerals in ferrocalcite and ankerite (CaFe_x_Mg_1-x_(CO_3_)_2_, 0≤x≤1) dissolve firstly and produce maximal dissolved amounts, while precipitation happens later. For example, calcite is dissolving from 0.0 km to 3.0 km with the maximal value of [Ca^2+^] obtained at depth of 0.8 km, and then precipitates with deeper depth than 3.0 km.

On effect of CO_2_ mole fraction, with the increasing CO_2_ mole fraction from 0.1% to 10.0% in carbonate system, the aqueous concentrations of metal species show a similar trend of increase firstly and then decrease. For example, dissolved amount of CaFe_0.7_Mg_0.3_(CO_3_)_2_ increases and reaches a maximum of 1.78 mmol·L^-1^ at a burial depth of 0.7 km with CO_2_ mole fraction at 0.1%, while 8.26 mmol·L^-1^ is obtained at the same depth of 0.7 km with 10.0% of CO_2_.

For the influence of overpressure generated during diagenesis, with the overpressure ranging from 36MPa to 83 MPa in calcite system, Δ[Ca^2+^] increases slightly from -2.24 mmol·L^-1^mmol·L^-1^ to -2.17 mmol·L^-1^mmol·L^-1^, indicating it is also a precipitation process at burial depth of 3.9 km where overpressure generated.
